# Calcium Handling by Endoplasmic Reticulum and Mitochondria in a Cell Model of Huntington’s Disease

**DOI:** 10.1371/currents.hd.37fcb1c9a27503dc845594ee4a7316c3

**Published:** 2016-01-06

**Authors:** Agnese De Mario, Chiara Scarlatti, Veronica Costiniti, Simona Primerano, Raffaele Lopreiato, Tito Calì, Marisa Brini, Marta Giacomello, Ernesto Carafoli

**Affiliations:** Biomedical Sciences, University of Padova, Padova, Italy; Venetian Institute for Molecular Medicine, Padua, Italy; Biomedical Sciences, University of Padova, Padova, Italy; Pediatric Hemato Oncology Clinic, Woman and Child Health, University of Padova, Italy; Biomedical Sciences, University of Padova, Padova, Italy; Biomedical Sciences, University of Padova, Padova, Italy; Biology, University of Padova and VIMM Padova, Italy; Biomedical Sciences, University of Padova, Padova, Italy; Venetian Institute of Molecular Medicine, Padova, Italy

## Abstract

Huntington disease (HD) is caused by the CAG (Q) expansion in exon 1 of the IT15 gene encoding a polyglutamine (poly-Q) stretch of the Huntingtin protein (Htt). In the wild type protein, the repeats specify a stretch of up 34 Q in the N-terminal portion of Htt. In the pathological protein (mHtt) the poly-Q tract is longer. Proteolytic cleavage of the protein liberates an N-terminal fragment containing the expanded poly-Q tract becomes harmful to cells, in particular to striatal neurons. The fragments cause the transcriptional dysfunction of genes that are essential for neuronal survival. Htt, however, could also have non-transcriptional effects, e.g. it could directly alter Ca2+ homeostasis and/or mitochondrial morphology and function. Ca2+ dyshomeostasis and mitochondrial dysfunction are considered important in the molecular aetiology of the disease. Here we have analyzed the effect of the overexpression of Htt fragments (18Q, wild type form, wtHtt and 150Q mutated form, mHtt) on Ca2+ homeostasis in striatal neuronal precursor cells (Q7/7). We have found that the transient overexpression of the Htt fragments increases Ca2+ transients in the mitochondria of cells stimulated with Ca2+-mobilizing agonists. The bulk Ca2+ transients in the cytosol were unaffected, but the Ca2+ content of the endoplasmic reticulum was significantly decreased in the case of mHtt expression. To rule out possible transcriptional effects due to the presence of mHtt, we have measured the mRNA level of a subunit of the respiratory chain complex II, whose expression is commonly altered in many HD models. No effects on the mRNA level was found suggesting that, in our experimental condition, transcriptional action of Htt is not occurring and that the effects on Ca2+ homeostasis were dependent to non-transcriptional mechanisms.

## INTRODUCTION

Huntington disease (HD) is a genetic neurodegenerative disorder characterized by choreiform movements, progressive cognitive decline and inexorable progression to death 15-20 years from the time of onset. Although the genetic cause of HD, Huntingtin (Htt), is expressed not only in neural cells but also in other tissues, the primary site affected in this pathology include several brain regions. Besides the cortex, cerebellum and thalamus, one of the hallmark of the disease is the loss of neuronal cells in the striatum (caudate and putamen), with selective damage to the GABAergic medium spiny neurons^1^. Molecularly, HD is caused by the CAG expansion in exon 1 of the IT15 gene encoding a polyglutamine stretch (polyQ) in the amino-terminal region of the Htt^2^. The polyQ tract begins at the 18th amino acid of Htt and contains 11–34 glutamine residues in unaffected individuals, but expands to various lengths in HD patients. The number of the CAG repeats in the gene inversely correlates with the age of onset^3^: a length ranging from 40 to 50 Qs is associated with adult onset, whereas expansions exceeding 60 Qs occur in the juvenile onset. Interestingly, even if Htt is ubiquitously expressed in human tissues, its mutation is specifically harmful to striatal neurons. The reason of this selective damage is not completely understood, reflecting the incomplete knowledge of the function of Htt itself. However, the protein is known to play an essential role during development, as the deletion of its gene is embryonic lethal^4^, and is claimed to participate in different cell functions like axonal transport, exocytosis and Ca^2+^ homeostasis^5^^,^ 6. Thus, Htt mutations could cause dysfunctions in one or more of these processes, contributing to HD aetiology. The extended polyQ tract is cleaved off by various caspases, calpain and other proteases. The cleaved off fragments have strong tendency to polymerize forming aggregates, whose role is debated. A possible damaging role could result from the sequestration of essential factors, e.g. transcription factors or calmodulin, whereas a positive role could be the binding and hence neutralization of monomeric Htt fragments, which are likely to be the damaging species. Pathological Htt (mHtt) fragments have been found in the nucleus^7^, where they may damage neurons by a transcriptional mechanism. Bioinformatics analysis that compared gene expression profiles (archived in public databases) in different HD cell models has highlighted the up-or downregulation of Ca^2+^ related genes^8^. It has also been reported that changes in the expression levels and activity of components of the Ca^2+^ handling toolkit^9^^,^ 10 and of the respiratory chain (ETC) occur in some HD models^11^^,^ 12 ^,^ 13.

Defective mitochondria, which are key players in the maintenance of intracellular Ca^2+^ homeostasis, have also been involved in the pathogenesis of HD. In particular, the involvement of ETC defects, more specifically of complex II, is supported by the finding that administration of the specific inhibitor of complex II 3-nitropropionic acid (3-NPA) induces a degeneration of rat striatal neurons that mimics that seen in the disease^14^. Even if mitochondrial dysfunction is considered important in the molecular aetiology of HD^15^^,^ 16 ^,^ 17, it is still not understood whether it results from transcriptional effects or whether non-transcriptional effects could also play a role. Transcriptional effect of mHtt have been repeatedly reported^18^^,^ 19 ^,^ 20 ^,^ 21, however non transcriptional effects of mHtt probably mediated by interactions with other proteins have also been claimed^22^^,^ 23 (and see Table I).

**Table 1:** Binding of the wt (wtHtt) and mutated (mtHtt) Htt to proteins including those of the Ca^2+^ handling system.

Protein name	Technique	mHtt wtHtt
Calretinin	Tandem affinity purification	mHtt
Calmodulin	Affinity chromatography	both
InsP3R	Co Immunoprecipitation assay	both
VDAC2	Yeast two-hybrid screening and mass spectrometry	wtHtt
PACSIN 1	Co Immunoprecipitation assays	both
HAP1	Yeast two-hybrid screening	both

Ca^2+^ dyshomeostasis is also frequently considered as a factor in the aetiology of HD. Ca^2+^ signaling is crucial for a number of neuronal activities and also for the development and maintenance of neuronal circuits^24^^,^ 25. The dysfunction of Ca^2+^ homeostasis associated to HD could also be due either to transcriptional or non-transcriptional defects. As an example, the increased binding of mHtt to the Inositol-1,4,5-trisphosphate receptor (InsP_3_R) has been related to a possible enhancement of Ca^2+^ release from the endoplasmic reticulum (ER)^26^: in principle, the increased exposure of mitochondria to Ca^2+^ released from the vicinal ER could exacerbate their dysfunction, even if a complex II defect would limit the functioning of the respiratory chain and consequently the ability of mitochondria to accumulate Ca^2+^ 27. However, the Htt effects on mitochondrial Ca^2+^ homeostasis appears to be more complicated, as increased mitochondrial Ca^2+^ uptake has been reported in other HD models^8^^,^ 9 ^,^ 15.

The aim of this work was to study the effects of the Htt fragments on Ca^2+^ homeostasis in a commonly used HD cell model, i.e. an immortalized striatal precursor cell line (Q7/7)^28^. Immortalization of these striatal precursors maintains most of the important processes of primary brain cells and has allowed the identification of a number of effects of the Htt fragments. We have previously shown that in these neuronal precursor cells Ca^2+^ transients could be generated by the stimulation with the InsP_3_ linked agonists, e.g. the purinergic agonist ATP, or bradykinin^10^. Here, we have transiently transfected the Q7/7 cells with a set of plasmids carrying the first exon of the IT15 gene, containing either 18 (wt, wtHtt) or 150 (mHtt) CAG repeats, and have monitored the changes of [Ca^2+^] with aequorin targeted to the mitochondrial matrix or to the cytoplasm. Ca^2+^ transients were induced by the InsP_3_-linked agonist ATP. The data have shown that the overexpression of either the 18Q or the 150Q Htt fragments enhanced mitochondrial Ca2+ transient with respect to controls, i.e. the Q7/7 cells only overexpressing the red fluorescent protein Kate and the Ca^2+^ probe aequorin. Consistent with the results of Fernandes and coworkers^61^, the expression of the fragments had no effect on the bulk cytosolic Ca^2+^ transients, whereas the ER Ca^2+^ content, evaluated by means of a probe specifically targeted to its lumen, showed a lower value in cells expressing the 150Q fragments.

A number of studies have reported transcriptional effects already occurring a few hours after the induction of mHtt expression^19^^,^ 29 ^,^ 30. Considering the long turnover times of mitochondrial proteins, e.g. the respiratory chain components^31^, the occurrence of transcriptional effects induced by our protocol on mitochondria was unlikely. We nevertheless decided to verify their possible occurrence. A thorough transcriptomic analysis such as that described in^32^ was beyond the scope of this article, thus we focused on a representative gene, subunit A of complex II (SDHA), whose expression was found altered in many HD model cells^27^^,^ 33. In our experimental protocol, no changes of the transcript for SDHA were detected, indicating that the overexpressed Htt fragments had no transcriptional effects and suggesting that they affected Ca^2+^ homeostasis by a non-transcriptional mechanism.

## MATERIALS AND METHODS

**Plasmids construction**

Wild-type (18Q) and mutant (150Q) cDNA of human IT15 exon 1 gene, encoding the N-terminal (1-90) region of the Htt protein, have been obtained by PCR amplification using as template the plasmid pIND-HD exon 1- EGFP 150Q (Wang, 1999) and the following primers, inserting the EcoRV and BamHI at the 5’- and 3’-ends, respectively: For (5’-CTCTAGATATCATGGCGACCCTGGAAAAGCTG-3’); Rev (5’-GTGGATCCGGTCGGTGCAGCGGCTCCTCAGC¬-3’). Since the intrinsic instability of CAG repeats, a series of PCR products of different size have been obtained, and the fragments carrying 18 and 150 Qs have been specifically selected and purified after agarose gel separation. Upon EcoRV/BamHI endonuclease digestion, DNA fragments have been cloned into the Kate-pcDNA3.1 plasmid digested with the same enzymes, giving recombinant vectors able to express in mammalian cells the Htt18Q- and Htt150Q-exon1 proteins fused at their C-terminus with the red fluorescent protein Kate. All the constructs have been finally verified by sequencing.

**Cell culture and transfection**

Clonal striatal cell lines established from E14 striatal primordial of WT-HdhQ7 littermate mouse embryos (Q7/7) were used^28^. The cells were grown in Dulbecco’s modified Eagle’s medium High Glucose (DMEM, Euroclone), supplemented with 10% fetal bovine serum (FBS, Euroclone), 100 U/ml penicillin and 100 mg/ml streptomycin (Euroclone), and maintained at the permissive temperature (33°C) in a humidified incubator with 5% CO_2_. 24 h before transfection, cells were seeded onto 13 mm (for aequorin measurements and RT-PCR experiments) or 24 mm (for FRET analysis) glass cover slips and allowed them to grow to 70–80% confluence.

For Ca^2+^ measurements and RT-PCR experiments, Q7/7 cells were co-transfected with the cDNA encoding the red fluorescent protein Kate, representing our control, or with a cDNA encoding for exon 1 of the Htt gene containing 18Q or 150Q (both fused with Kate) together with the cDNA coding for the aequorin Ca^2+^ probe targeted to the mitochondrial matrix (mtAEQ) or to the cytoplasm (cytAEQ) in a 1:2 ratio. For FRET analysis, Q7/7 were co-transfected with the red fluorescent protein Kate or 18Q, 150Q expressing plasmids with ERD1 (R.Y. Tsien, UCSD, CA)^34^ in a 1:2 ratio. Transfection was performed with the calcium-phosphate procedure, as previously described in^35^, or by using Lipofectamine (Invitrogen) according to the manufacturer’s instruction. Experiments were performed 48h after transfection. The expression and correct cellular localization of Htt fragments were analyzed by fluorescence procedures as reported in Fig.1.

**Aequorin Measurements**

Mitochondrial aequorin (mtAEQ) and cytosolic aequorin (cytAEQ) were reconstituted by incubating cells with 5 µM wt coelenterazine (Invitrogen) for 1 h in Krebs Ringer modified buffer (KRB, 125 mM NaCl, 5 mM KCl, 1 mM Na3PO4, 1 mM MgCl_2_, 20 mM HEPES, 5,5 mM glucose, pH 7.4, 37°C)-containing 1 mM CaCl_2_ and then transferred to the perfusion chamber of a purpose-built luminometer. Ca^2+^ measurements were started in the KRB medium added with 1 mM CaCl_2_, and after 30 sec 100 µM ATP (Sigma) was added as indicated in the Figs. 2, 3. All the experiments were terminated by cell lysis with 100 µM digitonin (Sigma) in a hypotonic Ca^2+^-rich solution (10 mM CaCl_2_ in H_2_O) to discharge the remaining reconstituted active aequorin pool. The light signal was collected and calibrated off-line into Ca^2+^ concentration values, using a computer algorithm based on the Ca^2+^ response curve of wt and low affinity aequorin as previously described^36^.

**ER Ca^2+^ measurement with the ERD1 Ca^2+^ probe**

Cells expressing the fluorescent probe ERD1 were analyzed using an inverted fluorescence microscope (Olympus lX-81) with an immersion oil objective 60× UPLAN FLN (NA 1.25; Olympus, Tokyo, Japan). Excitation light at 425 nm was produced by a monochromator. The emitted light was collected through a beam splitter (Multi Spec Micro-Imager; Optical Insights; emission filters 480 ± 15 and 545 ± 20 nm) and a 505 nm dichroic mirror. Images were collected by means of the Cell R imaging software (Olympus) and analyzed with ImageJ. FRET Ratio have been calculated according to the formula: YFP (background corrected)/CFP(background corrected).

**RNA isolation and reverse transcription PCR experiments**

mRNA for SDHA and for the isoforms of Ryanodine receptor (RyR) were detected by the RT-PCR. Transfected and untransfected cells were washed three times with cold phosphate-buffered saline and collected using TRIzol reagent (Invitrogen). Total RNA was extracted, according to the manufacturer’s protocol, and 1 µg of total RNA was reverse-transcribed using a Superscript III reverse transcriptase (Invitrogen). An amount of cDNA corresponding to 1–10 ng of total RNA was amplified in 25 µl of a mixture containing 12.5 µl of Platinum SYBR Green qPCR SuperMix-UGD (Invitrogen), 2 µl of primers mixture (2.5 µM each). The PCR cycling parameters were: 94 °C for 7 min, 45 cycles of 94 °C for 30 s, 55 °C for 30 s, and 72 °C for 15 s. The used primers have the following sequences: SDHA forward, AGGCTTGCGAGCTGCATT; reverse, AATGCCATCTCCAGTTGTCC; RyR1 forward, CGAGAGGCAGAACAAGGCAG; reverse, GGTCCTGTGTGAACTCGTCA; RyR2 forward, GGAAGAAAATGAAGCGGAAA; reverse, AGGGGCACAGATGTTCAGTC; RyR3 forward, GGCCAAGAACATCAGAGTGACTAA; reverse, TCACTTCTGCCCTGTCAGTTTC; GAPDH forward, CAAGGTCATCCATGACAACTTTG; reverse, GGGCCATCCACAGTCTTCTG.

**Statistical analysis**

All of the data are representative of at least ten different experiments unless otherwise indicated. Values are expressed as mean ± SEM. Statistical significance was determined using the Student’s t test. *p < 0.05; **p < 0.001; **** p<10-5, Student’s t-test.

## RESULTS

**Localization of Htt fragments in Q7/7 cells**

To study the effects of Htt fragments on intracellular Ca 2+ homeostasis plasmids carrying the cDNAs of exon 1 of Htt, coding for either 18Q or 150Q fused to the red fluorescent protein Kate were used. As a control we transfected cells with only the plasmid encoding the Kate fluorescent protein. Fig. 1 shows that the cells overexpressing only the Kate protein (Fig. 1A) and those overexpressing the 18Q Htt fragment (Fig. 1B) had homogeneous staining in the cytoplasm. However, aggregate-like structures with particularly high fluorescence intensity could be detected in some cells expressing either the 18Q (Fig. 1C) or the 150Q fragment (Fig. 1D,E), suggesting that the strong overexpression of wtHtt somehow favored the formation of aggregates, mimicking what occurs in case of mHtt. These aggregates were similar to those observed in other HD cell models^37^^,^ 38 ^,^ 39.

**Expression and subcellular localization of Htt fragments in Q7/7 cells:**
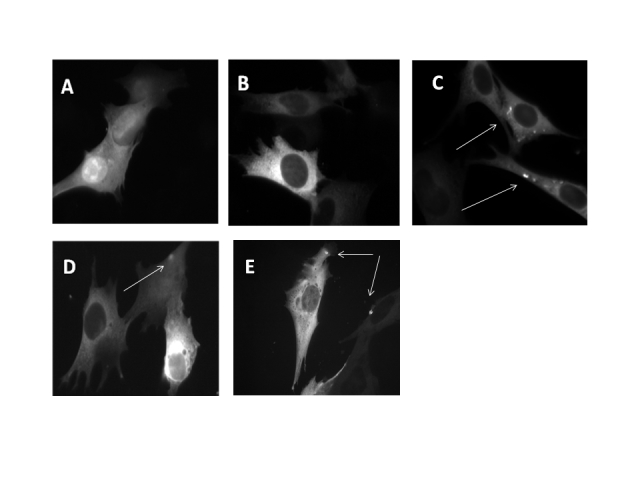
Fluorescence images of Q7/7 cells transfected with the cDNA of the fluorescent protein Kate (A) or of Htt exon 1 containing either 18Q (B,C) or 150Q (D,E). Some of the cells overexpressing 18Q fragments show the presence of aggregate like structures which are more frequently found in 150Q expressing cells (as indicated by arrows).

**Mitochondrial Ca^2+^ transients in cells overexpressing Htt fragments**

Impairment of various aspects of the Ca 2+ uptake/extrusion system of mitochondria has been repeatedly described in HD model cells^40^^,^ 41, but the issue is still controversial. We have analyzed the effects of Htt fragments on mitochondrial Ca 2+ handling by studying them in Q7/7 cells co-expressing the Ca 2+ sensitive probe aequorin targeted to the mitochondrial matrix^35^ along with Htt exon 1 carrying either 18Q or the 150Q repeats. The cells were then challenged with the purinergic InsP 3 -linked agonist ATP in the presence of extracellular Ca 2+ to induce Ca 2+ release from the ER as well as Ca 2+ entry from the extracellular medium. Fig. 2 shows that the Ca 2+ transients were significantly higher in the mitochondria of cells overexpressing either the 18Q (Fig. 2B) or 150Q (Fig. 2C) - containing fragments than in controls (Fig. 2A) (average of peak values, Fig. 2D: 82.98 ± 5.18 µM in control Q7/7 cells, n=40; 124.58 ± 6.71 µM in Q7/7 cells overexpressing the 18Q fragment, n=38 p<6.0410-6; 114.70 ± 6.24 in Q7/7 cells overexpressing the 150Q fragment, n=35 p<2.1*10-4). No significant difference was detected between 18Q and 150Q, suggesting that the observed effect on the mitochondrial Ca 2+ transient was unrelated to the length of the polyQ stretch.

**Mitochondrial Ca2+ handling in Q7/7 cells overexpressing Htt fragments:**
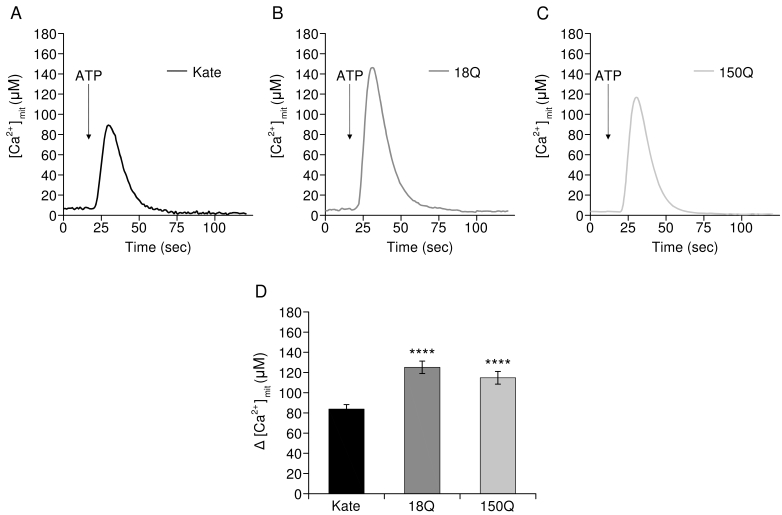
Representative traces of Q7/7 cells challenged with ATP expressing mitochondrial aequorin and: A) the Kate protein, B) 18Q fragment, C) 150Q fragment. D) Average of mitochondrial [Ca 2+ ] peaks (20 independent experiments; **** p<10-5, Student’s t-test).

**The Htt fragments affect the ER Ca^2+^ level but not cytosolic Ca^2+^ transients**

To understand whether the increased mitochondrial Ca2+ transients in 18Q and 150Q- overexpressing cells were linked to alterations of the global cytosolic Ca2+, the cells were transfected with cytAEQ^36^ and then challenged with ATP. As shown in Fig. 3 the overexpression of the Htt fragments (Fig. 3B, C) had no effect on the global cytosolic Ca2+ transients induced by the stimulation (average peak values, Fig. 3D: 1.99 ± 0.45 µM in control Q7/7 cells, n=22; 2.15 ± 0.19 µM in Q7/7 cells overexpressing the 18Q fragment, n=15; 1.95 ± 0.24 in Q7/7 cells overexpressing the 150Q fragment, n=15).

**Cytosolic Ca2+ transients are not affected by the overexpression of Htt fragments:**
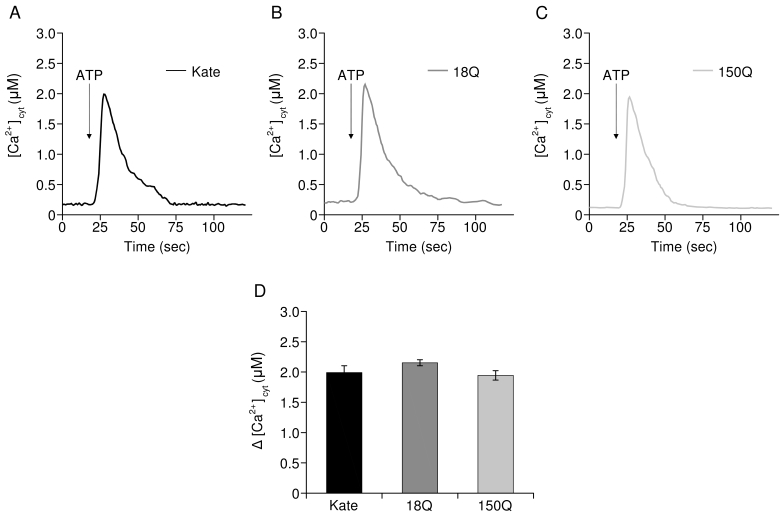
Representative traces of Q7/7 cells challenged with ATP expressing cytosolic aequorin and: A) the Kate protein, B) 18Q fragment, C) 150Q fragment. D) Average of cytosolic [Ca2+] peaks.

We therefore turned our attention to the main intracellular Ca^2+^ store, the ER: mitochondrial Ca^2+^ uptake senses Ca^2+^ released from the ER to generate microdomains of high Ca^2+^ concentration in close proximity of mitochondria^42^^,^ 43. We decided to measure the luminal Ca^2+^ content of the ER in the three Q7/7 cell populations (i.e. controls, 18Q, 150Q) to correlate the observed mitochondrial Ca^2+^ transients to possible changes in the ER Ca^2+^ levels. The ER-directed FRET based Cameleon ERD1^34^. This probe consists of two fluorescent proteins (a brighter and less pH sensitive version of YFP known as cpVenus and CFP) fused in tandem with a modified version of the Ca^2+^ binding protein Calmodulin. Binding of Ca^2+^ to the probe results in a conformational change of D1ER that brings together the two fluorophores allowing FRET to occur. Ca^2+^ concentration is therefore proportional to the increased FRET Ratio. Our experiments revealed that cells expressing the 150Q fragment had lower levels of Ca^2+^ in the ER than those expressing 18Q or the controls (Fig 4). These results are in line with the findings by Tang et al^26^, who had shown that mHtt interacts with the InsP_3_R to modulate its Ca^2+^ leak activity.

**mHtt fragments affect the ER Ca2+ level:**
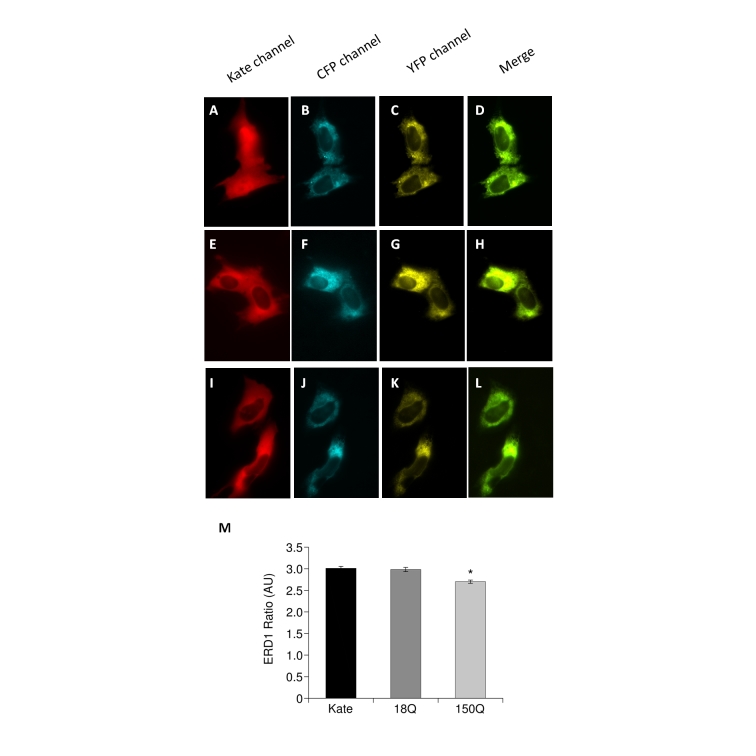
Panels A-D: Typical images of Q7/7 cells coexpressing Kate and the Ratiometric Ca2+ probe ERD1. Panels E-H: Typical images of Q7/7 cells coexpressing 18QKate and the Ratiometric Ca2+ probe ERD1. Panels I-L: Typical images of Q7/7 cells coexpressing 150QKate and the Ratiometric Ca2+ probe ERD1. Per each cell type, a representative image is reported for the two channels composing the FRET probe, CFP and YFP, as well as their merge. The Ratio value (proportional to the ER Ca2+ concentration) has been calculated as specified in the Material and Methods section and in^34^. Panel M: The histogram reports the Ratio values measured in resting conditions of Q7/7 cells coexpressing the three Kate fluorescent proteins and ERD1. The 150Q fragment displayed reduced levels of Ca2+ in the ER, with respect to 18Q or Kate. (Average of 3 independent experiments; * p<0.05, Student’s t-test).

**ER Ca^2+^ is released by InsP_3_R**

To support the idea that in our cell model, the alteration of mitochondrial and ER Ca^2+^ was related to action of Htt on InsP_3_R rather than on other (ER) Ca^2+^ release channels, i.e., the Ryanodine receptor (RyR), we explored the expression levels of the three isoforms of RyR (RyR1, RyR2 and RyR3) in Q7/7 cells. Interestingly, we found that these cells do not express any isoform of RyR (Fig. 5, cerebellar granule neurons (CGN) and Hela cells were used as positive and negative controls, respectively). Thus, Ca^2+^ was released from the ER of Q7/7 uniquely through the InsP_3_R.

**RT-PCR analysis of RyRs transcripts:**
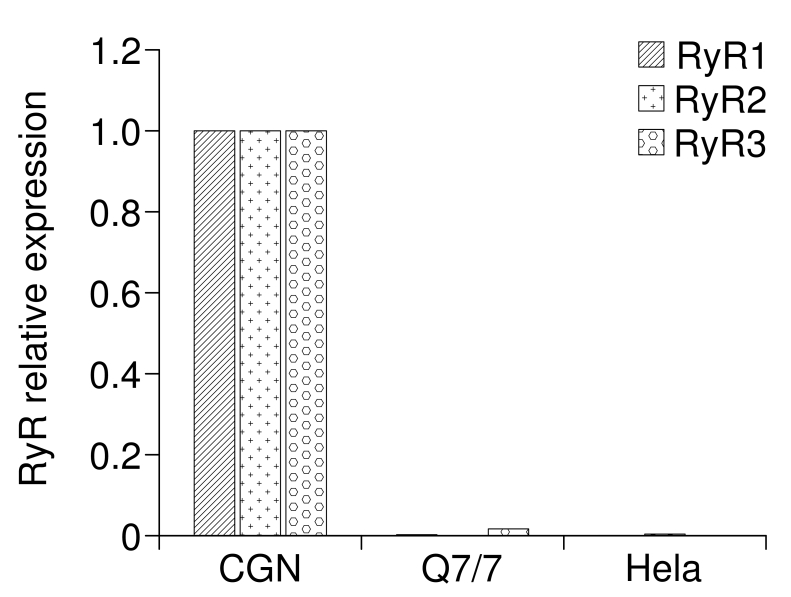
RT-PCR analysis of RyRs transcripts**.** Expression of RyRs in Q7/7 cells. Cerebellar granule neurons (CGN) and Hela cells were used as positive and negative control respectively. The results are normalized with respect to positive control.

**Overexpression of Htt fragments does not affect the transcription of SDHA**

Even if the protocol used was unlikely to produce transcriptional effects on mitochondrial membrane proteins; however others have been suggesting the possibility of early transcriptional defect in HD cell models^62^. To rule out the possibility of transcriptional effects we decided to verify if this was indeed so in the Q7/7 overexpressing the 18Q and 150Q fragments in our transient expression experiments. We focused on SDHA since its expression is commonly down regulated in HD model cells^12^^,^ 13 ^,^ 44. RT-PCR analysis (Fig. 6) revealed that the overexpression of the Htt fragments failed to alter the levels of SDHA transcripts. As mentioned above, this was not surprising, as most of the mitochondrial proteins have a low turnover rate^31^. Therefore, the effects of the Htt fragments on Ca^2+^ homeostasis in the mitochondria here described are likely due to a non transcriptional mechanism.

**RT-PCR analysis of the subunit A of the mitochondrial respiratory chain (SDHA) transcript:**
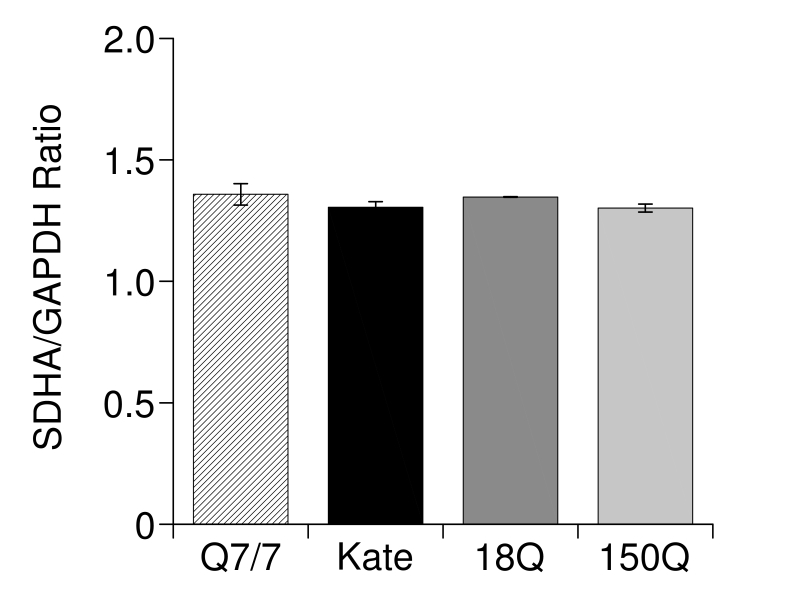
RT-PCR analysis of the subunit A of the mitochondrial respiratory chain (SDHA) transcript **.** Expression of SDHA in Q7/7 untransfected (Q7/7) or transfected with the protein Kate, and the 18Q or 150Q fragments. These experiments have been performed after checking the quality of the retro-transcribed cDNA on agarose gel (not shown). The results are normalized respect to the housekeeping gene GAPDH.

## DISCUSSION

The involvement of mitochondrial defects in the molecular aetiology of HD was suggested for the first time more than ten years ago, based on the evidence that 3-NPA, an inhibitor of complex II of the respiratory chain, was per se able to induce Htt-like symptoms^14^^,^ 45 ^,^ 46. A number of reports have then described defects in the morphology of mitochondria in HD model cells^15^^,^ 19 ^,^ 47 ^,^ 48 ^,^ 49. Impaired respiratory chain activity^50^^,^ 51 and abnormalities of mitochondrial Ca^2+^ handling have been described in most of the experimental HD models currently in use^9^^,^ 41 ^,^ 52, reinforcing the idea of a role for mitochondrial defects and Ca^2+^ dyshomeostasis in the development of the disease.

Opinions on the topic are divergent: some studies have claimed that mitochondria from HD model cells have increased propensity to depolarize and are more susceptible to Ca^2+^ overload, i.e. to the induction of the permeability transition (PTP)^33^^,^ 53 ^,^ 54. Other studies have reported opposite results, concluding that mHtt would instead lower the probability of PTP opening^55^^,^ 56. These discrepancies could be due to the use of isolated mitochondria, removed from the influence of the physiological context of intact cells, or to the use of stable cell lines which could develop adaptive mechanisms to compensate for the derangement of specific intracellular processes^17^^,^ 41. However, experimental outcomes obtained in primary striatal neurons carrying a pathogenic mHtt with 128Q show that PTP inhibitors significantly reduced NMDA-induced PTP opening^61^.

In previous work on stable clones of Q7/7 cells^10^, we had found that cells stably expressing a Htt stretch of 111Q were characterized by higher sensitivity of mitochondria to Ca^2+^ overload, and were more susceptible to the mitochondrial permeability transition, as also found by other groups^9^^,^ 33. Notably, the cell lines used in our previous work displayed extensive transcriptional changes, especially in the levels of InsP_3_-controlling enzymes which were mostly downregulated^10^. Thus, the alterations of Ca^2+^ homeostasis in the Q cell experimental model could be explained in two alternative ways: either by transcriptional effects of the stably expressed mHtt, or by a compensatory process that counterbalanced the enhanced response to stress of the mitochondria by altering the levels of intracellular Ca^2+^ pools. Accordingly, we and others had found that the amount of Ca^2+^ released upon stimulation of the cells with a cell permeable derivative of InsP_3_ 10 or with the inhibitor of the ER SERCA pump cyclopiazionic acid^15^ was higher in cells expressing the mutated than in the wt counterparts.

This work has explored the direct effects of mHtt on Ca^2+^ homeostasis by using the wt neuronal striatal precursors Q7/7 transiently expressing the N terminal portion of Htt, either in a wt form (18Q) or carrying a pathologically extended polyQ stretch (150Q)). It had been shown by others that the expression of the N terminal portion of Htt is sufficient to cause acute neuronal toxicity^57^. The overexpression of the N terminal Htt fragments has allowed us to specifically explore the contribution of this portion of the protein to the production of Ca^2+^ handling defects observed in the presence of the pathogenic Htt forms.

Consistently with our previous published results comparing Q7/Q7 and Q111/Q111 immortalized striatal cell lines^10^, and also with a study of cytosolic Ca^2+^ handling in primary striatal neurons from a murine model of HD^61^ we have found no changes in the bulk cytosolic Ca^2+^ transients.

Further, in agreement with reports on isolated mitochondria^41^^,^ 55 ^,^ 56, we have detected higher Ca^2+^ peaks in these organells upon stimulation of cells expressing either the 150Q or 18Q exon 1 of Htt. Since cytosolic Ca^2+^ was not affected, the results were likely due to a direct action of the Htt fragments on the mitochondrial Ca^2+^ handling machinery or on the Ca^2+^ toolkit of the ER, that is responsible for the generation of Ca^2+^ hotspots in the proximity of mitochondria. The evidence that in our experiments both wtHtt and mHtt increased mitochondrial Ca^2+^ uptake, may suggest that the control of the mitochondrial functions involved in the sensing of the ER linked Ca^2+^ hotspots could be a physiological function of Htt per se, not specifically of mHtt. In this context, it is interesting that Htt has been found associated to the outer mitochondria membrane (OMM) by means of its N terminus^33^^,^ 58. Alternatively, the effects observed with wtHtt could be due to the fact that the presence of large amounts of it in the cells due to its overexpression could mimic the effects of mHtt, as suggested by the finding that some cells with large expression of wtHtt displayed aggregates (Fig.1C).

As to the ER Ca^2+^ handling, we have found decreased ER Ca^2+^ levels specifically in the case of mtHtt. As previously claimed, Htt interacts with the Htt associated protein (HAP1A) to form a complex with the InsP_3_R, promoting its opening^26^: i.e., the mutated form of Htt enhances the Ca^2+^ release through InsP_3_R. Recent evidence underlines the importance of the specific transfer of Ca^2+^ to mitochondria by InsP_3_R for cell bioenergetics^59^. The Insp_3_R generates mitochondrial Ca^2+^ hotspots on the OMM surface, that drive Ca^2+^ uptake in the mitochondrial matrix^43^^,^ 60. No studies have so far performed on the role of the mutated form of Htt on the transfer of Ca^2+^ between ER and mitochondria. Further studies on this aspect would be necessary to shed light on the mechanisms underlying Ca^2+^ dyshomeostasis in HD.

## Competing Interests

The authors declare that there are no conflicts of interest.
